# Giant Cell Lesions of the Craniofacial Bones

**DOI:** 10.1007/s12105-014-0589-6

**Published:** 2014-11-20

**Authors:** Adrienne M. Flanagan, Paul M. Speight

**Affiliations:** 1 UCL Cancer Institute, 72 Huntley Street, London, UK; 2 School of Clinical Dentistry, University of Sheffield, Sheffield, UK

**Keywords:** Fibrous Dysplasia, Giant Cell Tumor, Aneurysmal Bone Cyst, Vide Infra, Noonan Syndrome

## Introduction

Multinucleate giant cells of one type or another are commonly encountered in oral and maxillofacial lesions. These include the common occurrence of foreign-body-type giant cells in some reactive lesions, and giant cells associated with granulomatous inflammation as a consequence of infection. However in these cases the giant cells do not represent the primary pathology. In this brief review, we will focus on lesions that may arise in the jaws and in which osteoclasts-like giant cells are a characteristic or defining feature. The classification of this group of lesions remains problematic, because for some lesions, for example, central giant cell granulomas of the jaw, the true nature or cause of the lesions has not been established. In other cases, molecular pathology is now beginning to unravel the pathogenesis of these lesions, and also their relationships to each other. We will not include a discussion of the pathology of hyperparathyroidism except to emphasize that when an osteoclast-rich tumor is encountered within the jaw bones, consideration should be given to the exclusion of hyperparathyroidism. This is usually straightforward based on radiology and appropriate serology.

## Aneurysmal Bone Cyst

### General Features

Aneurysmal bone cyst (ABC) is an osteolytic tumor arising in the intramedullary cavity [1]. There are two variants, primary ABC which is characterized by a *USP6* gene rearrangement [2, 3], and secondary ABC which may arise as a reactive process in association with almost any other benign or, less commonly, malignant bone tumor [4]. ABC present as radiolucent lesions with a characteristic ballooning of the cortex, and are most commonly encountered in individuals <30 years of age, although the diagnosis has been confirmed by fluorescent in situ hybridization (FISH) using a break-apart probe for *USP6*, presenting in a 57 year old [2, 5].

Although lesions may recur, the treatment of choice is a conservative procedure, most commonly curettage.

### Histopathology

**Fig. 1:**
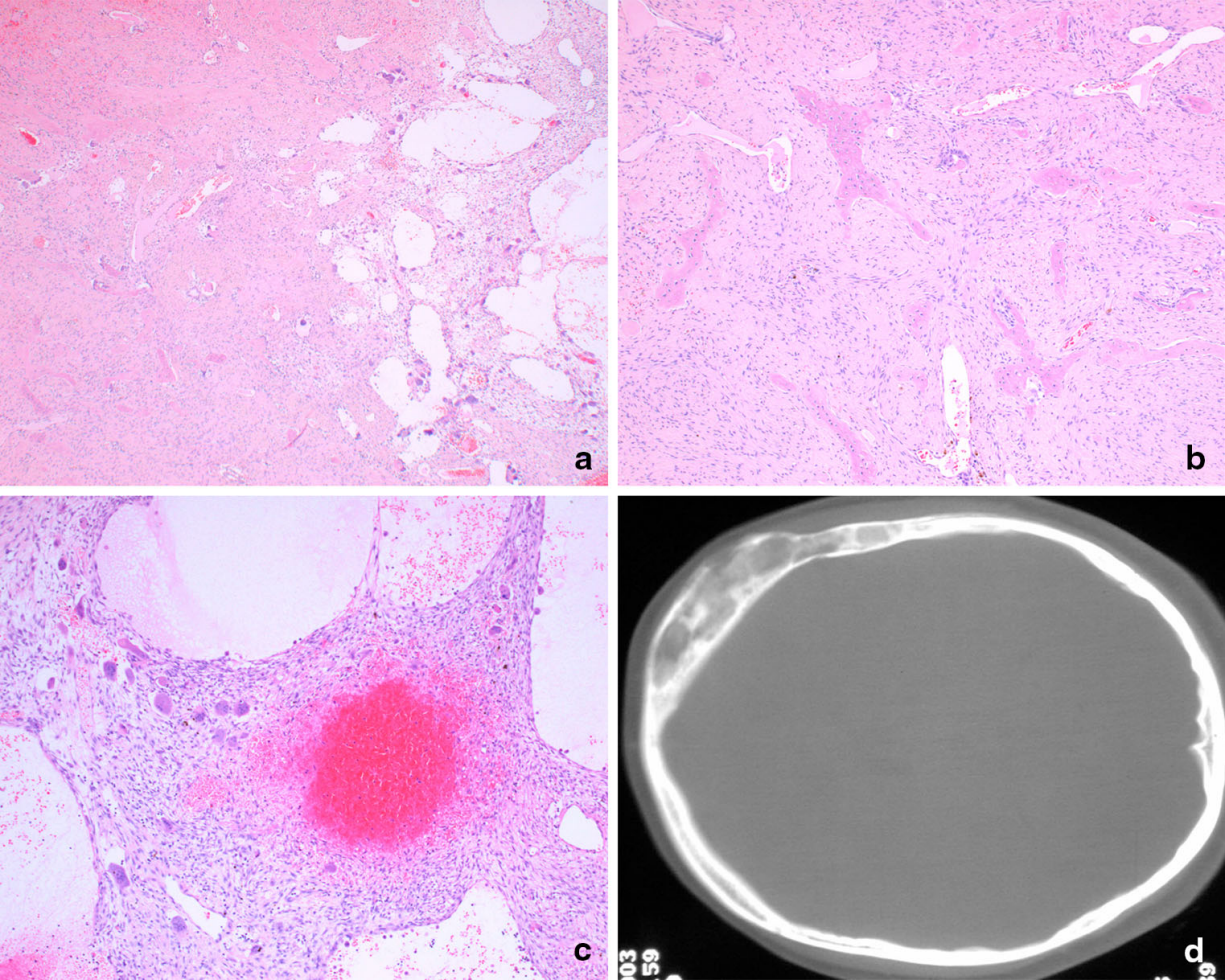
Light photomicrographs and X-ray of fibrous dysplasia harboring a *GNAS* mutation (R201C) with secondary ABC change. **a** A low power magnification of a fibro-osseous lesion merging with a cystic lesion. **b** Bony trabeculae, not lined by osteoblasts, embedded in the bland spindle cells. **c** Cystic spaces, the wall of which are composed of spindle cell in which numerous osteoclasts are present. **d** X-ray of skull showing osteolytic lesion with cortical break-though

ABC is characterized microscopically by a spectrum of features which are present to varying degrees [1, 6]: the tumor may be dominated by cystic spaces which are often blood-filled surrounded by thin septa, in which there may be osteoid deposition, lined by spindle-shaped cells which do not express endothelial cell markers (CD31, CD34 and ERG negative by immunohistochemistry). Solid areas may dominate in some tumors. Osteoclasts are often ‘lined up’ within the septa and can protrude into the cystic spaces. The osteoid may have a blue hue (blue bone), which is characteristic of this tumor. ABC are also composed to a lesser or greater extent by solid areas of monotonous spindle cells, which can be mitotically active, although the figures are normal in configuration. The tumor cells do not show cytological atypia and necrosis is not generally a feature. The amount of osteoid deposition is highly variable but can be quite extensive. Distinguishing primary and secondary ABC can be impossible purely on histological grounds in the absence of sampling of the primary tumor, such as fibrous dysplasia, osteoblastoma, chondromyxoid fibroma, giant cell tumor of bone, and conventional cartilaginous tumors, amongst others (Fig. 1; Table 1).

**Table 1:** Genetic alterations in osteoclast-rich tumors

Diagnosis	Genetic alteration—type	Specific alteration
Sporadic		
Aneurysmal bone cyst (primary)	Rearrangement/fusion gene	t(16;17)(q22;p13) [3, 4]
Giant cell granuloma (peripheral and central)	No known	
Chondroblastoma	Substitution	*H3F3B* and *H3F3A* p.Lys36Met (p.K36M) [11]
Giant cell tumour of bone	Substitution	*H3F3A* p.Gly34Trp (p.G34W) [11]
Fibrous dysplasia	Substitution	*GNAS1* [9]
Germline		
Cherubism	Substitution, occasional deletion reported	*SH3BP2* [40]
Noonan syndrome	Substitution	*PTPN11*, *SOS1*, *RAF1* [29]
Leopard syndrome	Substitution	*PTPN11* [34, 36]
Craniofacial cutaneous syndrome	Substitution	*BRAF*, *MAP2K1* [37]
Neurofibromatosis type 1	Substitution, indels	*NF1* [25, 38]

### Molecular Pathology

Approximately 75 % of primary ABC harbor a balanced chromosomal translocation involving *USP6* on 17p13 [4, 5]. A variety of fusion partners including CDH11, ZNF9, COL1A1, TRAP150, and OMD have been reported [3, 4]. The spindle cells in ABC harbor the genetic alteration and not the osteoclasts or their precursors [4], and experimental evidence suggests that the oncogenic impact of the *USP6* rearrangement results in alteration of cell migration and cytokinesis [7]. Although rare in the craniofacial bones, the characteristic fusion gene involving (6:17)(p21;p13) has been detected by cytogenetics in an intranasal tumor in a 6 year old [8]. Secondary ABC does not harbor a *USP6* alteration, although the detection of a genetic aberration characteristic of the primary tumor, such as *GNAS* R201 alterations involving R201H (~57 %), R201C (~38 %), and Q227L (~5 %) in fibrous dysplasia [9] (Fig. 1), a *GRM1* alteration in chondromyxoid fibroma [10], and H3.3 alterations in giant cell tumor of bone and chondroblastoma [11], can help in reaching a diagnosis.

It is noteworthy that *USP6* rearrangements have also been detected in close to 90 % of nodular fasciitis [5, 12], a soft tissue tumor, often a reaction to trauma, that resolves spontaneously. The *USP6* alteration has also been detected in some cases diagnosed as myositis ossificans, and it has been suggested that these would be better classified as soft tissue ABCs [12]. It is noteworthy that whereas *MYH9*, on chromosome 22q12.3, is the common fusion partner (65 % of cases) with *USP6* in nodular fasciitis, it has not been reported in ABC [5, 12].

## Chondroblastoma

### General Features

Chondroblastoma is classified as a benign intramedullary cartilaginous tumor, accounting for approximately 1 % of all primary bone tumors. The tumors occur at the ends of the long bone, and at the apophysis, and can present in the immature skeleton [1, 6].

There are only a small number of reports of chondroblastoma occurring in the bones of the craniofacial region with the temporal bone being most commonly affected. The largest series reported included 30 cases collected from multiple institutions by Bertoni et al. [13], and there are also other case reports and small series [14]. Whereas the majority of chondroblastoma of the long bones present in the second and early third decade, those reported in the craniofacial region present more commonly later—in the third and fifth decade. The treatment of choice is curettage.

### Histopathology

**Fig. 2:**
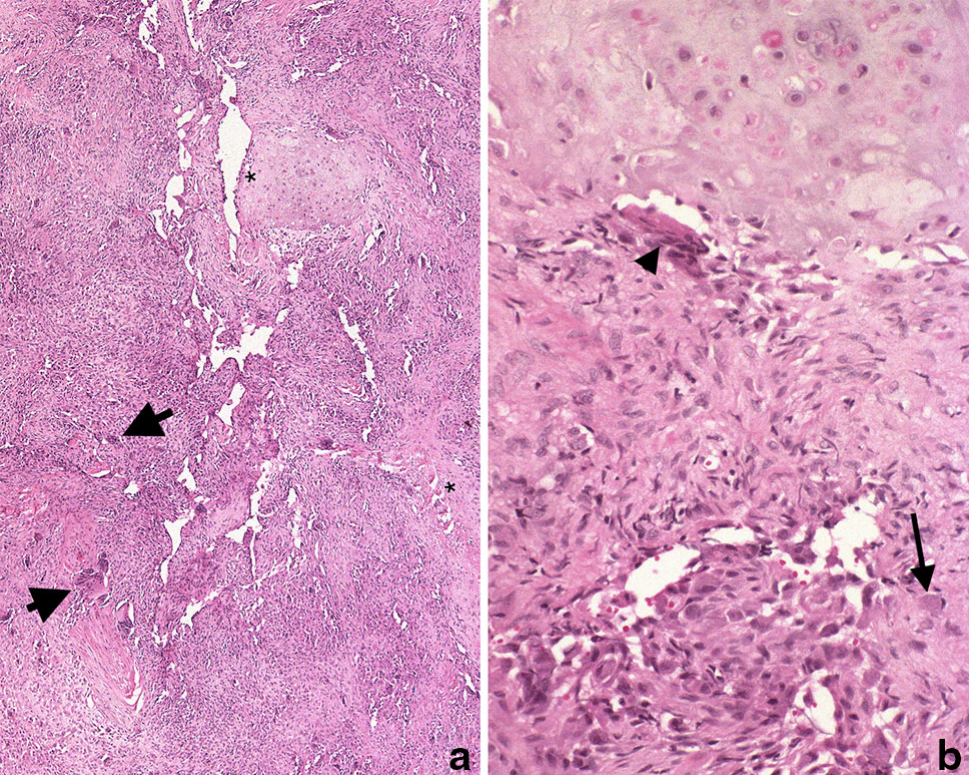
Haematoxylin and eosin-stained sections of a chondroblastoma. **a** A low-power view showing sheets of a ‘monotonous’ cell population in which scattered larger cells (osteoclasts—*arrows*) are noted. Islands of cartilaginous matrix *asterisk* are present. **b** The cartilaginous component sharply demarcated from spindle and round cells without atypia in which there is conspicuous eosinophilic cytoplasm (*arrow*) and osteoclasts (*arrowhead*). Courtesy of Lester D. R. Thompson

Chondroblastomas in the bones of the craniofacial region have the same histological features as those at other sites. The tumor has a biphasic appearance comprising chondroid-rich, and osteoclast-rich components. The islands or sheets of cartilage are generally sharply demarcated from the osteoclast-rich areas, and the proportion of the two components varies considerable from tumor to tumor (Fig. 2). This can result in difficulties in reaching a diagnosis, particularly on a needle core biopsy, with the differential diagnosis being determined by the component that is sampled. The chondroid area of chondroblastoma shares similarities with other primary cartilaginous tumors such as chondromyxoid fibroma, conventional and mesenchymal chondrosarcoma and chordoma, whereas diagnoses including GCG and ABC would be considered if the osteoclast-rich area were sampled (vide infra). However, pericellular calcification is a well-recognised feature of chondroblastoma, and can be a helpful in arriving at a diagnosis, as this is not characteristic of other cartilaginous tumors, although in chondroblastoma, this calcification process can be focal.

The multinucleate ‘giant cells’ in chondroblastoma are considered to be osteoclasts: in this tumor, just as in giant cell tumor of bone, the osteoclasts are abnormally large and contain as many as 50 or more nuclei. They can be so numerous that the intervening tumor mononuclear cells may be overlooked. The mononuclear cell population has a characteristic grooved nucleus; the nuclear chromatin is smooth, and mitotic figures, although present in small numbers, are normal. Host bone entrapment is not seen, although there can be extensive endosteal erosion at the tumor-host bone interface [1, 6].

Tumor cells in the chondroid component of chondroblastoma express S100, and cytokeratin expression is also a feature of this tumor type [15], but these findings are of little value in distinguishing chondroblastoma from other lesions with histological similarities, because of the lack of sensitivity and specificity of these markers. Brachyury expression is valuable in distinguishing cartilaginous tumors from chordoma [16].

### Molecular Pathology

Through whole genome sequencing, using massively parallel sequencing technology of 6 chondroblastomas, we identified the presence of recurrent H3.3 alterations. An extension study revealed that 95 % (73/77) of tumors with typical features of chondroblastoma harbored a p.Lys36 Met (p.K36 M) substitution in either the replication-independent histone variants, H3.3, which encode *H3F3A* or the *H3F3B* genes [11]. These are present on chromosome 1 and 17 respectively and the 2 genes share identical protein sequences but have different exonic and intronic DNA sequences. The *H3F3B* gene was more commonly affected than the *H3F3A* gene in chondroblastoma but other than these alterations, the genomes revealed no other recurrent aberrations and there was a relatively low number of somatic changes. Copy number and rearrangement analysis showed that the tumors overall were diploid and had low numbers of structural changes [11]. To date, there have been no reports of H3.3 alterations in either chondroblastomas of gnathic bones, or bones of the skull.

Cystic change (secondary ABC) is well recognized as occurring in association with many primary bone tumors, including chondroblastoma, and can represent a major element of the tumor. We have found that the p.K36M is detected in a number of cases where this represents a major element of the tumor and has aided in reaching the correct diagnosis (unpublished).

The p.K36M mutations are mutually exclusive with the H3.3 mutations reported in giant cell tumor of bone at extra-gnathic sites (vide infra) [11]. Furthermore, the alterations are mutually exclusive with *USP6* rearrangements, which are detected in ~75 % of primary ABC (vide supra), and were not detected in chondromyxoid fibromas, which are characterized by a complex alteration in chromosome 6 involving the glutamate receptor gene *GRM1* in 80 % of cases. This results in over-expression of GRM1 through the recombining of several partner genes, through promoter swapping and gene fusion events [10]. H3.3 alterations are also reported in only 1/75 conventional or dedifferentiated cartilaginous tumors and are mutually exclusive of *isocitrate dehydrogenase* (*IDH*) *type 1* or *IDH2* substitutions which occur in 60 % of conventional or dedifferentiated cartilaginous tumors [11, 17]). Therefore H3.3 K36M alterations represent a valuable adjunct in reaching a diagnosis of chondroblastoma, and allow chondroblastoma to be distinguished from other cartilaginous and osteoclast-rich lesions (Table 1). However, it is not possible to rely entirely on the genetic alteration when making a diagnosis as no single biomarker is 100 % specific or sensitive and H3.3 alterations are also present rarely in osteosarcoma. Specifically, one osteosarcoma with a H3.3 p.G34W, and 2 osteosarcomas with a H3.3 p.G34R (one in *H3F3A* and one in *H3F3B*) have been reported among 110 osteosarcomas [11, 18].

## Giant Cell Granuloma

### Central Giant Cell Granuloma

#### General Features

**Fig. 3:**
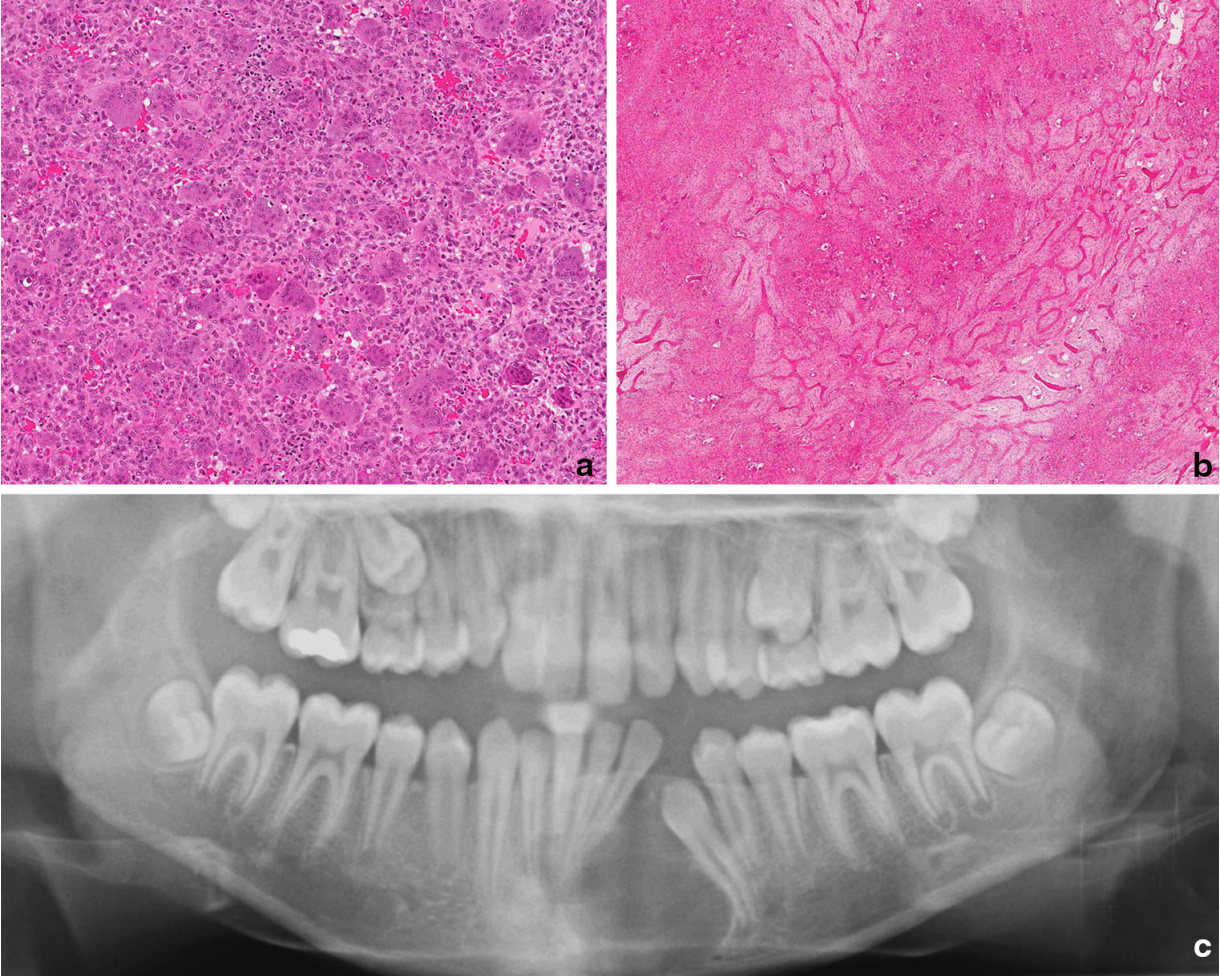
Central giant cell granuloma. **a** Accumulations of multinucleated giant cells embedded in a cellular stroma composed predominantly of plump mononuclear cells. These are primarily osteoblasts and mononuclear osteoclast precursors. The lesion is vascular with areas of red cell extravasation. **b** In many lesions the focal accumulations of osteoclasts are separated by septa of woven bone. **c** Radiology shows a well demarcated and in places corticated radiolucency. Lesions may be large and often displace the adjacent teeth

Central giant cell granuloma (GCG) is an intramedullary bone lesion involving the mandible and maxilla, the former being affected more frequently. Although they present over a wide age range, they occur more frequently under the age of 20. They are classified clinically as non-aggressive and aggressive lesions, the former being slow-growing, generally painless lesions without evidence of tooth resorption and cortical perforation. In contrast, the aggressive lesion is painful with or without paresthesia and is associated with tooth resorption and cortical perforation: it also has a higher risk of local recurrence following curettage which is the treatment of choice (Fig. 3) [6].

Giant cell granuloma are sited most commonly in the anterior jaw. They can be solitary or involve the jaw more extensively. Although gnathic GCG shares similarities with conventional giant cell tumor of extra-gnathic sites, they are considered to be separate entities on the basis that there are sufficient differences in the histology and behavior (vide infra) to warrant this distinction. The recent finding of H3.3 alteration in 92 % of giant cell tumors of bone and not in GCG supports this (vide infra) [11].

##### Histopathology

Giant cell granuloma is composed of a monotonous mononuclear cell population in which low numbers of mitotic figures are noted. The characteristic feature is a variable numbers of giant cells, which have been shown to be osteoclasts [19]. These may contain up to 20 nuclei but are generally not as large as those seen in conventional extra-gnathic giant cell tumors of bone. The osteoclasts are commonly grouped in clusters associated with thin-walled vascular channels, with evidence of hemorrhage and haemosiderin deposition (Fig. 3). The stromal cells comprise a mixture of spindled fibroblasts and tartrate-resistant acid phosphatase-positive polygonal cells which represent osteoclast precursors [19], and variable numbers of osteoblasts [20]. In GCG the osteoclasts are usually more widely distributed, and do not occur as dense collections with almost no detectable intervening stroma as seen in conventional extra-gnathic GCG. The tumors are contained by a thin rim of peripheral bone, and a limited amount of osteoid and woven bone can be seen within the main tumor mass. The histological features of solitary gnathic GCG are similar to those seen in osteoclast-rich lesions of the jaw, which occur on the genetic background of Noonan syndrome, Neurofibromatosis type 1 (NF1), Cherubism and craniofacial cutaneous syndrome (vide infra) [6].

Immunohistochemistry is unhelpful in reaching a diagnosis. The osteoclasts and their precursors are immunoreactive for CD45 and CD68, and tartrate-resistant acid phosphatase.

### Molecular Pathology

The majority of GCG of the jaw represents sporadic disease and is not characterized by a recurrent genetic alteration, and specifically does not harbor *SH3BP2* alterations reported in Cherubism (vide infra) [21, 22]. Similarities have been made between GCG of the small tubular bones of the hand and feet, and GCG of jaw. Recently, Agaram et al. [23] reported that 8/9 GCG of the small tubular bones of the hand and feet harbored a *USP6* rearrangement, the genetic hallmark of the ABC, and therefore argued that such tumors should be classified as primary ABC similar to those in other bones harboring this genetic alteration. They also used FISH to look for *USP6* rearrangements in 8 gnathic GCG but did not detect any such alterations.

Recently we reported that 92 % of conventional extra-gnathic GCT harbor a H3.3 alteration, which always occur in the *H3F3A* gene and involve p.Gly34 (p.G34). The vast majority result in a substitution p.Gly34Trp (p.G34W) and much less commonly in p.Gly34Leu (p.G34L) [11]. As in the chondroblastoma genomes (vide supra), there were no other recurrent alterations, and there was a paucity of somatic changes with no copy number changes, and rearrangements other than two regions of loss of heterozygosity. In view of the overlapping features of solitary central gnathic GCG and conventional extra-gnathic GCT, we screened 78 of the former for H3.3 p.G34 substitutions but failed to detect any. It is possible that some of these lesions were related to Noonan syndrome, NF1, Cherubism, or craniofacial cutaneous syndrome as the mutations associated with these syndromes were not sought, but the absence of the H3.3 alterations in so many cases implies that GCG is distinct from GCT and represents another disease for which a genetic alteration remains to be identified.

### Noonan Syndrome and Neurofibromatosis Type 1

A minority of cases of central GCG of the jaw arises on the background of Noonan syndrome, and Neurofibromatosis type 1 (NF1) (vide infra) [24–27]. In such cases, the lesions are often multiple and behave in an aggressive manner, and present in young patients <20 years of age. The jaw lesions may be the presenting symptom and/or sign in an individual with one of these syndromes and therefore a thorough clinical examination for other more common stigmata of these entities, such as *café au lait* spots, should be undertaken. Even in the absence of definitive signs of Noonan syndrome or NF1, screening for the relevant germline alterations should be considered, particularly if there is more than one jaw lesion, and if the lesion is extensive. This is because phenotypes of these syndromes may be very mild [28]. However, clotting disorders and cardiac defects in Noonan syndrome are important to detect as they can be managed pro-actively. The jaw lesions in Noonan syndrome and NF1 can be so extensive that they can mimic Cherubism, and therefore this diagnosis should be borne in mind and excluded (vide infra) [25, 29, 30].

Noonan syndrome and NF1 are among the most commonly encountered germline alterations, with an incidence of 1 in 2,500—1 in 3,000 of the population. Noonan syndrome, a complex clinical genetic disorder, is inherited as an autosomal dominant trait caused by alterations in *PTPN11*, *SOS1*, *RAF1*, *KRAS*, *NRAS*, and *BRAF* genes and characterized by short stature, craniofacial dysmorphism, short neck with webbing, deformity of the sternum, cardiac and clotting anomalies, and cryptorchidism [31]. The most common germline alterations involve *PTPN11* (~50 %), *SOS1* (10–15 %), and *RAF1* (5–10 %); with *KRAS* mutations only occurring in 2 % of those affected. The severity and the spectrum of the phenotypic changes are significant, with some individuals having almost no clinical stigmata of the disease. A small proportion of individuals with Noonan syndrome also exhibit multiple gnathic GCG previously reported as Noonan-like/multiple GCG, but this phenotype is now recognized to be allelic with Noonan syndrome [32], as mutations in *PTPN11*, *SOS1*, and *RAF1* have been reported in this syndrome [33, 34]. LEOPARD syndrome is also allelic with Noonan syndrome and is associated with two recurrent *PTPN11* mutations in exons 7 (Tyr279Cys) and 12 (Thr468Met), although other less common alterations are also seen. We have reported on an individual with LEOPARD syndrome and multiple GCG of the jaw caused by alterations in *PTPN11* [35, 36]. Very occasional patients with craniofacial cutaneous syndrome, classified as a RASopathy (vide infra), and multiple giant cell tumors of the jaw syndrome have also been reported with *BRAF* or *MAP2K1* mutations [37].

Neurofibromatosis type 1, Noonan syndrome, LEOPARD syndrome and craniofacial cutaneous syndrome are considered RASopathies as a consequence of germline mutations in genes encoding specific proteins of the RAS/mitogen-activated protein kinase (MAPK) pathway [27, 30, 35]. The activation of this pathway during development results in patients with these four disorders exhibiting overlapping phenotypes: Noonan syndrome and NF1 are both associated with freckling/*café au lait* spots, and occasionally multiple GCG of the jaw, in addition to dysmorphic craniofacial features, congenital cardiac defects, skin abnormalities, varying degrees of intellectual disability, and increased risk of malignancies (acute leukemia). It has on some occasions been difficult to distinguish these two syndromes and some individuals were therefore classified as having neurofibromatosis-Noonan syndrome. However, there is now evidence that this syndrome is allelic to NF1 in most patients [25, 38].

### Cherubism

Cherubism is a rare benign disease characterized by symmetric enlargement of the jaw and is limited to the mandible and maxillary bones [1]. It is inherited as an autosomal dominant trait, and caused by mutations in the SH3-domain binding protein 2 (*SH3BP2),* sited in 4p16.3, resulting in variable penetrance and expressivity. Approximately 50 % appear to arise as de novo mutations. Males and females are affected equally, and there is no ethnic predilection. The lesion presents in children up to the age of 6 [36, 39].

The disease obtained its name from the apparent upward gaze of the eyes, as seen in Baroque paintings of Cherubs by Ruben, resulting from displacement of the orbits and retraction of the eyelids as a consequence of lesional tissue involving the floor of the orbit. Involvement of the optic nerve and proptosis may also occur, and lymphadenopathy can also be seen in children. The disease stabilizes at puberty and if the phenotype is not severe may largely resolve but the dysmorphism may persist in those severely affected.

**Fig. 4:**
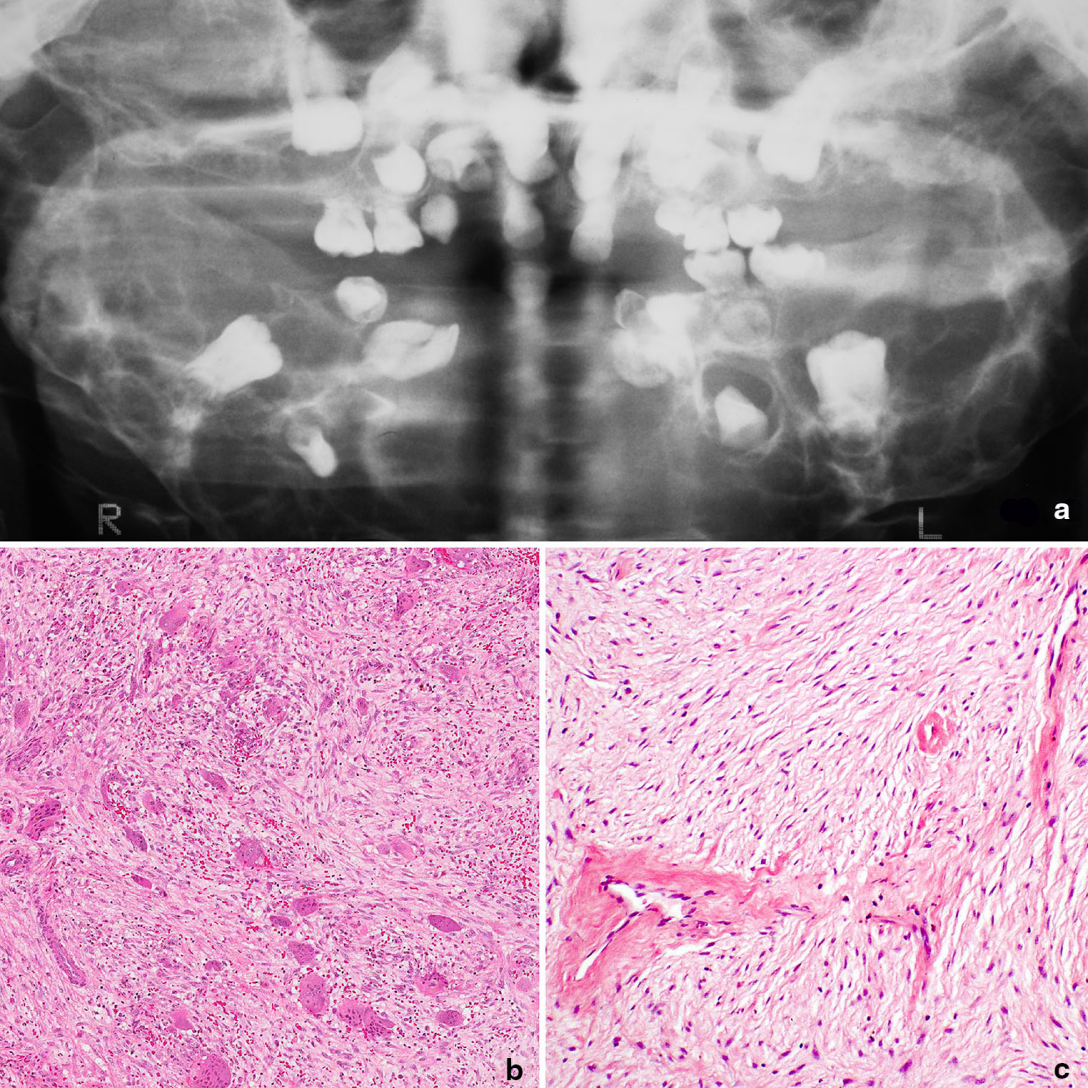
Cherubism, radiology and histology. **a** Radiology showing an extreme case of cherubism with extensive bilateral replacement of the jaws with radiolucent fibrous tissue. The lesions are typically multilocular giving a characteristic “soap bubble” appearance, and ‘free floating teeth’. **b** The giant cells and areas of haemorrhage are similar to those seen in giant cell granuloma, although in cherubism the stroma tends to be composed of fibrous connective tissue with fibroblasts arranged in sheets with a slight storiform or fascicular pattern. **c** In some areas osteoclasts are not conspicuous and blood vessels are prominent, and in some cases may be surrounded by a cuff of hyalinized collagen (Courtesy of John Wright)

The radiological appearance of the established disease is characteristic with massive expansion of the jaws associated with multilocular radiolucencies (Fig. 4), although in the early stages changes may be seen only at the mandibular angles. The histology reveals an osteoclast and spindle cell lesion, with overlapping features with those of GCG of the jaw (Fig. 4).

In contrast to Noonan syndrome and NF1, Cherubism is a non-complex genetic disease, which only affects the jaw and is not associated with other stigmata such as freckling or *café au lait* spots. A major distinguishing factor between Cherubism and these other syndromes is that the jaw lesion is always symmetric and always presents early (<6 years old) [36]. In the absence of *SH3BP2* mutation in an individual considered to have Cherubism, genetic screening for mutations in genes implicated in Noonan syndrome (*PTPN11*, *SOS1*, *RAF1*, *KRAS*, *NRAS*, and *BRAF*), *NF1*, and craniofacial cutaneous syndrome (*BRAF*, *MAP2K1*) should be undertaken [31]. As with all osteoclast-rich lesions, hyperparathyroidism should be excluded in the first instance.

SH3BP2 is an adaptor protein encoded by 13 exons, and is involved in signal transduction by forming complexes with other proteins. The majority of the mutations occur in exon 9 within a 6 amino acid sequence (RSPPDG), which is a proline-rich domain proximal to the SH2 domain of SH3BP2, although alterations have also been reported in exon 3 and 4, and mutations in exon 3 appear to be associated with a severe phenotype [40]. Other more rare mutations have been more reported more recently.

A mouse model has been developed to study Cherubism and has revealed that the Cherubism mutation (Pro416Arg) results in bone resorption, and increased levels of TNF-α, a cytokine known to increase osteoclast recruitment [41]. The mutations result in activation of certain signaling pathways as a result of *SH3BP2* stabilization through inhibition of tankyrase-mediated destruction of the protein. However, this did not explain why in humans the disease is restricted to the jaw [42]. Recently, Yoshitaka et al. [43] reported that macrophages harboring the *SH2BP3* alterations are hyper-responsive to pathogen-associated and damage-associated molecular patterns, both of which activate Toll-like receptors, and that the disease in mice is rescued by depletion of toll-like receptors. They speculate that the presence of a large amount of toll-like ligands, such as oral bacteria, present during development of the jaw bones cause the anatomical-specific development of human Cherubism lesions. A recent report shows that an anti-TNF-α antagonist (Etanercept) can prevent or ameliorate the disease progression in Cherubism mice [43].

The treatment of Cherubism is determined by the severity of the disease and surgery may be considered. Radiotherapy is not generally advised.
